# Gestational weight gain in the REVAMP pregnancy cohort in Western India: Comparison with international and national references

**DOI:** 10.3389/fmed.2022.1022990

**Published:** 2022-10-05

**Authors:** Kamini Dangat, Sanjay Gupte, Girija Wagh, Sanjay Lalwani, Karuna Randhir, Shweta Madiwale, Hemlata Pisal, Vrushali Kadam, Shridevi Gundu, Nomita Chandhiok, Bharati Kulkarni, Sadhana Joshi, Caroline Fall, Harshpal Singh Sachdev

**Affiliations:** ^1^Mother and Child Health, Interactive Research School for Health Affairs, Bharati Vidyapeeth (Deemed to be University), Pune, India; ^2^Gupte Hospital and Research Centre, Pune, India; ^3^Department of Obstetrics and Gynecology, Bharati Medical College and Hospital, Bharati Vidyapeeth (Deemed to be University), Pune, India; ^4^Department of Pediatrics, Bharati Medical College and Hospital, Bharati Vidyapeeth (Deemed to be University), Pune, India; ^5^Division of Reproductive, Biology, Maternal and Child Health (RBMCH) and Nutrition, Indian Council of Medical Research, New Delhi, India; ^6^MRC Lifecourse Epidemiology Unit, University of Southampton, Southampton, United Kingdom; ^7^Department of Pediatrics and Clinical Epidemiology, Sitaram Bhartia Institute of Science and Research, New Delhi, India

**Keywords:** body mass index, gestational weight gain, pregnancy, weight gain, weight gain curves

## Abstract

**Objective:**

To determine the trimester specific gestational weight gain (GWG) in a population of pregnant women from Western India and compare it with the Intergrowth–21st international and an Indian reference (GARBH–Ini cohort—Group for Advanced Research on BirtH outcomes).

**Study design:**

A prospective longitudinal observational study was undertaken in Pune, West India and data for gestational weight gain was collected [the REVAMP study (Research Exploring Various Aspects and Mechanisms in Preeclampsia)]. Generalized Additive Models for Location, Scale and Shape method (GAMLSS model) were used to create GWG centile curves according to gestational age, stratified by BMI at recruitment (*n* = 640) and compared with Intergrowth-21st reference and GARBH–Ini cohort. Multivariable regression analysis was used to evaluate the relationship between GWG and antenatal risk factors.

**Results:**

The median GWG was 1.68, 5.80, 7.06, and 11.56 kg at gestational ages 18, 26, 30, and 40 weeks, respectively. In our study, pregnant women gained less weight throughout pregnancy compared to Intergrowth-21st study, but more weight compared to the GARBH–Ini cohort centile curves in all the BMI categories. GWG in overweight/obese women (BMI ≥ 25) was significantly lower (<0.001) as compared to underweight (BMI < 18.5), or normal weight women (BMI ≥ 18.5 and <25). The median GWG at 40 weeks in underweight, normal and overweight/obese women was 13.18, 11.74, and 10.48 kg, respectively. Higher maternal BMI, older maternal age, higher parity and higher hemoglobin concentrations were associated with lower GWG, while taller maternal height was associated with greater GWG.

**Conclusion:**

GWG of Indian women is lower than the prescriptive standards of the Intergrowth charts.

## Introduction

Appropriate gestational weight gain (GWG) is essential for optimal fetal growth and birth outcome (1, 2). It is also a measure of maternal nutrition status (3, 4). Suboptimal GWG is associated with unfavorable delivery outcomes (5, 6) including an increased risk of intrauterine growth restriction, low birth weight, and preterm birth (7, 8). Excessive GWG is associated with an increased risk of gestational diabetes, gestational hypertension, preeclampsia, preterm birth, cesarean delivery, macrosomia, infant mortality, postpartum weight retention, and childhood obesity (8–12). Recent reports indicate a high prevalence of inadequate gestational weight gain in South Asia, in particular in India (13, 14). Appropriate GWG is usually evaluated in comparison to two international standards – those of the Institute of Medicine (IOM) and International Fetal and Newborn Growth-21st (Intergrowth-21st) (9, 15).

Studies from Asian countries have evaluated the total GWG until the end of pregnancy (5, 16–23). However, this does not indicate weight gain during different trimesters of gestation (24). Based on the comparison of total and trimester specific GWG, it has been suggested that the IOM standards are not appropriate for Asian women, who are shorter or thinner than the population used to construct these references (25–27).

A multi-ethnic, international standard of GWG, known as Intergrowth-21st, derived from data collected in eight countries, including India offers a prescriptive GWG reference chart (9). This study selected healthy women who were at low risk of adverse maternal and perinatal outcomes and provided centiles of GWG for each week of gestation. In India, maternal malnutrition is highly prevalent (28) and there is a high burden of inadequate GWG (13). Despite these challenges, and the association between GWG and optimal fetal growth, there are only few studies in Asia that have explored weight gain in different trimesters of pregnancy and compared against global references (Intergrowth-21st) (2, 29). In India, there is only one study, from Haryana, that has recently established a GWG reference for gestational age. This study demonstrates that pregnant women from the GARBH-Ini cohort (Group for Advanced Research on BirtH outcomes) gained less weight during early pregnancy (at 18 weeks) when compared with the Intergrowth–21st reference (24). This study questions the use of western estimates to identify an appropriate GWG. The related editorial emphasizes a need for establishing local references (state level) for GWG (30). India is a vast country with substantial regional differences in diet and other determinants of GWG. It is therefore useful to examine GWG in various populations across India, which could inform country specific guidelines, and be of use for the clinicians for better monitoring of GWG.

The current study examines GWG in a population of pregnant women attending ante-natal clinics in two hospitals in Western India and compares the weight gain across different periods of gestation with Intergrowth–21st reference and GARBH–Ini cohort. We also compare the centile curves across various BMI categories.

## Materials and methods

### Study site and population selection

The current study is a part of the Indian Council of Medical Research (ICMR) funded Center for Advanced Research project on “Investigating mechanisms leading to preeclampsia” at IRSHA, Bharati Vidyapeeth University, Pune (5/7/1069/13-RCH), which has established a cohort of pregnant women who were followed from early pregnancy until delivery [the REVAMP study (Research Exploring Various Aspects and Mechanisms in Preeclampsia)].

This study was initiated in March 2017 in the city of Pune, in Maharashtra State, India, at two urban hospitals -Bharati Hospital, and Gupte Hospital. The primary objective of this study was to examine the associations of maternal LCPUFA (Long-chain polyunsaturated fatty acids) and one carbon micronutrient status in early gestation with clinical outcome in preeclampsia and to understand the operative biochemical and molecular mechanisms. The protocol of REVAMP study has been previously published (31). In brief, pregnant women planning to give birth in Bharati and Gupte hospitals were recruited at their first antenatal visit (11–14 weeks’ gestation) and followed up subsequently at 18–22 weeks, 26–28 weeks, and at delivery. These time points were dependent on the methods of the primary study (REVAMP). The World Health Organization (32) recommends an ultrasound scan <24 weeks of gestation as part of the routine antenatal care, mid-trimester anomaly scan at 18–22 weeks of gestation is more optimal timing for it (33). According to Diabetes in Pregnancy study Group India (DIPSI) criteria, testing for GDM detection should be done at 24–28 weeks of gestation (34, 35). Hence, 18–22 and 26–28 weeks, was selected as the two most common antenatal visits for the pregnant women also keeping in mind the logistic and financial constraints.

### Study participants

This study used data from all the participants (*n* = 1154) who enrolled between March 2017 and March 2022 in the REVAMP cohort. The study analysis included only those women who: (1) visited the hospitals for antenatal care <14 weeks’ gestation; (2) were aged 18–45 years; (3) had singleton pregnancies; and (4) were free of chronic diseases (*n* = 1096). The study aimed to recruit 100 women with preeclampsia and 200 normotensive women from early pregnancy, to give 87% power to detect a difference of the same magnitude when alpha is kept at 0.05 (36). To get a sample size of 100 women with preeclampsia we followed 1096 women longitudinally across pregnancy.

**FIGURE 1:**
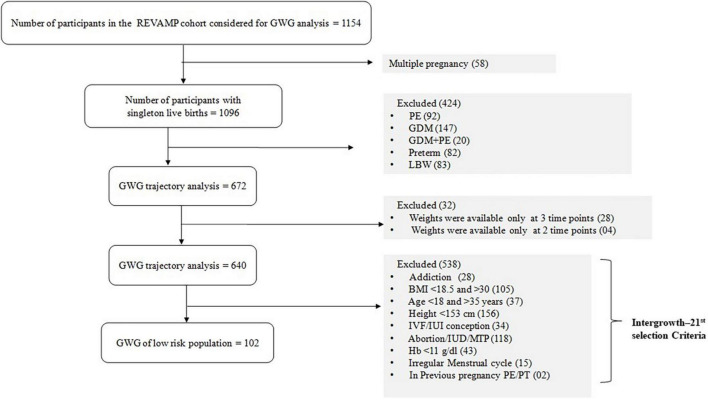
Flow diagram of the selection of the study population from REVAMP pregnancy cohort for gestational weight gain. PE, preeclampsia; GDM, Gestational Diabetes Mellitus; LBW, Low Birth Weight; PT, pre-term; GWG, gestational weight gain; BMI, body mass index; IVF, *in vitro* fertilization; IUI, intrauterine insemination; Hb, hemoglobin.

In order to obtain a ‘healthy’ sub-set, women with adverse pregnancy outcomes including gestational diabetes, hypertensive disorders, preterm births and low birth weight, were excluded from the study, leaving 672. Amongst these 672 women, information on GWG was not available for 32 women (out of the 4 visits of the study, 28 women had only 3 weight measurements and 4 women had only 2 weight measurements) and were excluded to generate smoothed centile curves of maternal weight across pregnancy. This resulted in a primary analytic sample of 640 participants for GWG trajectory analysis (Figure 1). In addition, we also selected a low-risk population, analogous to the Intergrowth-21st inclusion criteria (Supplementary Table 1) which included 15.9% (102/640) participants in our cohort.

### Data collection

#### Assessment of gestational age and anthropometric data

Gestational age was determined by last menstrual period (LMP) date, unless it differed from the gestation derived from the crown rump length at the first ultrasound scan (11–14 weeks) by > ± 7 days (18.6%), in which case the latter was used. Maternal weight (kg) at four time points (11–14, 18–22, and 26–28 weeks, and before delivery) was measured using calibrated digital weighing scales to the nearest 0.1 kg, at each antenatal visit (in both the hospitals). Height (cm) was measured once at the time of enrollment using to the nearest 0.1 cm using a stadiometer. Before the weight measurement, pregnant women were asked to take off heavy clothes, hand bags and shoes. Maternal weight and height were measured twice and the average of two reading was used for analysis.

#### Socio-demographic and clinical information

The socio-demographic details (socio-economic status, education) and clinical information (menstrual, obstetric data, parity, gestation, and mode of delivery), past and family history, were collected by a team of research assistants using a pre-tested questionnaire at the time of recruitment. All research assistants received on-site training prior to data collection. Socioeconomic status (SES) was recorded using the Standard of Living Index (SLI), a method developed by the International Institute for Population Sciences, Mumbai and used in India’s National Family Health Survey 2 (37). A questionnaire is used to collect data on family size, household assets, household amenities (toilets and source of drinking water), and ownership of land and livestock, from which a score is generated. At the time of recruitment hemoglobin levels were measured using venous blood sample at both hospitals on a fully automated hematology analyzer.

#### Assessment of gestational weight gain

We did not have pre-pregnancy weight for the participants in REVAMP, and so first trimester weight (11–14 weeks) was the baseline weight. Total weight gain was calculated by subtracting the 11–14 week weight from maternal weight on admission for labor, prior to delivery of the baby (38). Trimester specific GWG was determined by subtracting the measured weight in the first trimester from the measured weight at each subsequent prenatal visit. The recommended amount of GWG varies based on pre-pregnancy body mass index (BMI) of the women. The BMI grouping in the current study was based on IOM 2009 guidelines (15). BMI at recruitment was calculated as weight (kg)/height (m)^2^ and categorized was into three groups, underweight (<18.5), normal weight (18.5–24.9), and overweight/obese (≥25).

#### Assessment of physical activity and dietary score

Physical activity (1 month recall) was recorded at 11–14 weeks of gestation using a physical activity questionnaire that was broadly categorized by intensity (light/moderate/heavy). A daily score was calculated where higher scores indicate more activity.

Pregnant women were interviewed with a food frequency questionnaire at the same time point to estimate the frequency of consumption of foods identified using “Nutritive Values of Indian Foods” (39). All pregnant women had to indicate the frequency of each food consumed during the last 1 month for which scores were calculated.

### Funding, ethical approval and informed consent

This project was funded by the Indian Council of Medical Research (ICMR), New Delhi, India (5/7/1069/13-RCH dated 31-03-2017). The study for both the hospitals was approved by the Institutional Ethics Committee, Bharati Vidyapeeth Deemed University, Pune (IEC/2015/37, dated 03.10.2015). Written informed consent was obtained from each study participant. If the participant was illiterate or could not sign, then verbal/oral consent was taken and thumb impressions were obtained in the presence of an impartial witness who signed the consent document.

### Statistical analysis

Baseline characteristics (socio-demographic and clinical) of the study population were represented as median [interquartile range; IQR]. Categorical variables were expressed as number (n) and percent (%). We constructed the 3rd, 10, 25, 50, 75, 90, and 97th percentiles of GWG from 18 to 40 weeks of gestation by using the Lambda-Mu-Sigma (LMS) method *via* the GAMLSS model (Generalized Additive Models for Location, Scale and Shape package; version 5.1–6) in the R software (R version 4.1.2) (40).

The mean weight gain (mu), and sigma parameters of the Box-Cox Power Exponential distribution using cubic splines with five degrees of freedom were modeled against gestational age. For assessment of goodness of fit, smoothed centiles of GWG by gestational age were constructed and a visual inspection of the overall model fit was evaluated by comparing empirical centiles to the fitted centiles, using quantile–quantile plots of residuals, and plot of fitted z–scores across gestational ages.

Subsequently, we performed a comparison that was based on the 3rd, 5, 25, 50, 75, 95, and 97th GWG centiles at different gestational ages of the participants of this study with the International (Intergrowth-21st) and the National study curves (GARBH–Ini cohort). Similarly, we also compared centiles in the low-risk population group. We applied a multilevel linear regression analysis with antenatal risk factors (maternal age, BMI at the time of recruitment, height, parity, socio-economic status, family type, cooking fuel, occupation, drinking water, type of house and hemoglobin) as independent variables and GWG as the dependent variable. ANOVA was used to compare the mean GWG and BMI between the different BMI groups of first trimester BMI.

## Results

Table 1 summarizes the sociodemographic and clinical details of the study cohort. The median age of women in the cohort was 28 years (IQR, 25–31 years) and gestation at the first antenatal visit was 12.3 weeks (12–13 weeks). At enrollment majority of the study participants were in the normal weight category (59%), 10% of women were underweight and 31% were overweight or obese. The vast majority (90%) belonged to ‘upper’ socio-economic status. Overall, 40% of the women were graduates and 29% of the women were postgraduates. At baseline recruitment, the median hemoglobin level was 11.7 (g/dl) (IQR, 10.8–12.4 g/dl).

**TABLE 1:** Sociodemographic and clinical characteristics of the participants enrolled in the REVAMP cohort Pune, India (*n* = 640).

Sociodemographic characteristics	Median (IQR) or number (%)
Age (years)	28 (25–31)
Gestational age at enrollment (weeks)	12.3 (12–13)
**Body mass index (kg/m^2^) at enrollment Number (%)**	
Underweight (BMI < 18.5)	66 (10.3)
Normal weight (BMI ≥ 18.5 and <25)	375 (58.6)
Overweight (BMI ≥ 25)	156 (24.4)
Obese	43 (6.7)
Hemoglobin (g/dl) at recruitment	11.7 (10.8–12.4)
Height (cm)	155.3 (151.0–160.0)
**Socioeconomic status—Number (%)**	
Upper class	573 (89.5)
Upper middle class	64 (10)
Lower middle class	3 (0.5)
**Parity—Number (%)**	
0	365 (57)
1	252 (39.4)
2	23 (3.6)
**Level of education—Number (%)**	
Illiterate	1 (0.2)
Literate or primary school	2 (0.3)
Middle school (SSC)	71 (11.1)
High school	103 (16.1)
Graduate	258 (40.3)
Post-graduate	187 (29.2)
Vocational	14 (2.2)
No information	4 (0.6)
**Occupation—Number (%)**	
Professional	201 (31.4)
Semi-professional	43 (6.7)
Skilled	5 (0.8)
Semi-skilled	18 (2.8)
Unskilled	4 (0.6)
Others	3 (0.5)
Farmer	4 (0.6)
House-wife	354 (55.3)
No information	8 (1.3)
**Religion practiced in the household—Number (%)**	
Hindu	598 (93.4)
Muslim	19 (3.0)
Buddhist	8 (1.3)
Jain	8 (1.3)
Undetermined –no information	6 (1.1)

### Gestational weight gain centile curves across pregnancy

**FIGURE 2:**
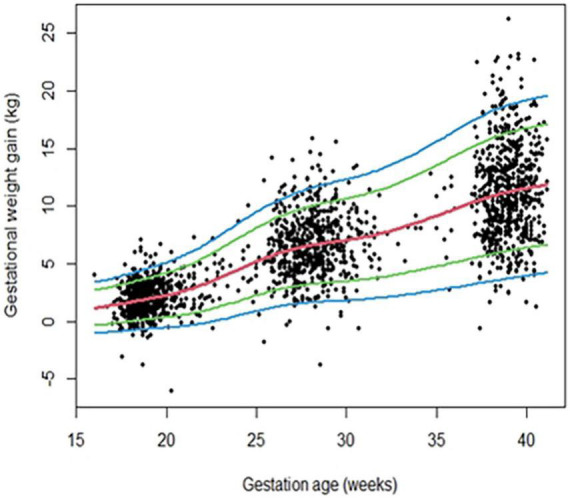
Gestational weight gain (GWG) pattern in the primary analytic sample (*n* = 640) of REVAMP cohort. Centile curves were generated for GWG using the GAMLSS.

Figure 2 depicts centile curves of gestational weight gain according to gestational age (18–40 weeks). Table 2, shows 3rd, 5, 25, 50, 75, 95, and 97th smoothed percentiles (estimated values), across pregnancy. The smoothed centile curves showed that the median GWG (kg) was 1.68, 5.80, 7.06, and 11.56 kg at gestational ages of 18, 26, 30, and 40 weeks, respectively (Table 2). Cumulative GWG between subjects was less in early pregnancy (18 weeks: IQR 0.80–2.57 kg) as compared to later gestational age (40 weeks: IQR 8.84–14.3 kg). The GWG 3rd, 5, 25, 50, 75, 95, and 97th centile at different periods of gestation for the low-risk group are tabulated in Supplementary Table 2.

**TABLE 2:** GWG distribution (in kg) across pregnancy in the REVAMP cohort, Pune, India (primary analytic sample; *n* = 640).

Gestational age (weeks)	*N*	Centiles for GWG (kg)						
		3rd	5th	25th	50th	75th	95th	97th
16	02	−1.02	−0.74	0.41	1.20	2.01	3.15	3.43
17	56	−0.93	−0.64	0.58	1.41	2.26	3.46	3.76
18	276	−0.79	−0.48	0.8	1.68	2.57	3.84	4.15
19	197	−0.65	−0.32	1.04	1.97	2.92	4.27	4.60
20	54	−0.55	−0.19	1.27	2.28	3.3	4.76	5.12
21	26	−0.41	−0.03	1.57	2.67	3.77	5.36	5.75
22	15	−0.19	0.24	1.98	3.18	4.39	6.12	6.55
23	06	0.15	0.61	2.51	3.82	5.15	7.04	7.50
24	10	0.52	1.02	3.09	4.52	5.96	8.02	8.52
25	25	0.90	1.44	3.66	5.20	6.75	8.97	9.51
26	102	1.23	1.81	4.17	5.80	7.45	9.80	10.37
27	157	1.47	2.07	4.54	6.25	7.97	10.43	11.03
28	169	1.63	2.26	4.82	6.60	8.38	10.93	11.56
29	94	1.73	2.37	5.01	6.84	8.68	11.32	11.96
30	43	1.80	2.46	5.18	7.06	8.95	11.66	12.32
31	21	1.91	2.59	5.39	7.33	9.28	12.07	12.75
32	02	2.07	2.77	5.67	7.68	9.69	12.58	13.28
33	04	2.27	3.00	6.02	8.11	10.21	13.22	13.95
34	02	2.50	3.27	6.43	8.62	10.82	13.98	14.74
35	09	2.73	3.54	6.87	9.18	11.49	14.81	15.62
36	01	2.97	3.82	7.33	9.75	12.19	15.68	16.53
37	88	3.21	4.10	7.77	10.32	12.87	16.53	17.42
38	216	3.46	4.39	8.19	10.83	13.48	17.27	18.20
39	218	3.72	4.66	8.55	11.25	13.96	17.84	18.79
40	113	3.95	4.91	8.84	11.56	14.3	18.22	19.18
41	06	4.18	5.14	9.10	11.85	14.61	18.56	19.52
42	02	4.41	5.38	9.37	12.14	14.91	18.89	19.86

The GAMLSS was used to calculate centiles.

### Gestational weight gain centile curves for participants across different body mass index categories

**FIGURE 3:**
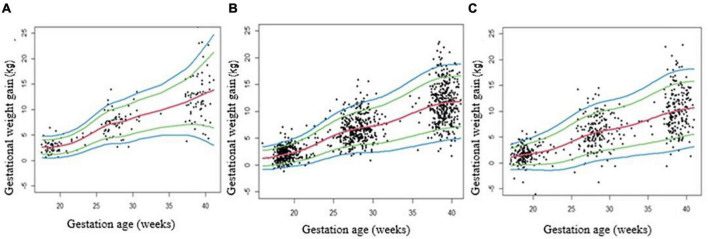
Gestational weight gain (GWG) centile curves for underweight **(A)**, normal weight **(B)** and overweight/obese **(C)** pregnancies. Centile curves were generated for GWG using the GAMLSS. **A:** Underweight (BMI < 18.5), **B:** Normal (BMI ≥ 18.5 and <25), and **C:** Overweight/obese (BMI ≥ 25).

The graphical representations of GWG centiles across pregnancy for underweight (*n* = 66), normal weight (*n* = 375), and overweight/obese (*n* = 199) pregnant women groups are shown in Figures 3A–C. The median values for GWG at 18 weeks were 2.62 kg in underweight women, 1.71 kg in normal weight and 1.40 kg in overweight/obese women. At 40 weeks of gestation the median value was 13.18 kg, in underweight women, 11.74 kg, in normal weight, and 10.48 kg in overweight/obese women (Table 3). GWG in overweight/obese women was significantly lower as compared to underweight or normal weight women (Supplementary Table 3).

**TABLE 3:** Percentiles of cumulative GWG (in kg) at various gestational ages (in weeks) among different first trimester BMI groups.

Gestational age (weeks)	3rd centile			5th centile			10th centile			50th centile			90th centile			95th centile			97th centile		
	Uw	N	Ow/Ob	Uw	N	Ow/Ob	Uw	N	Ow/Ob	Uw	N	Ow/Ob	Uw	N	Ow/Ob	Uw	N	Ow/Ob	Uw	N	Ow/Ob
16	0.66	–0.85	–1.43	0.92	–0.59	–1.15	1.31	–0.18	–0.72	2.71	1.27	0.81	4.1	2.71	2.32	4.5	3.11	2.75	4.76	3.38	3.03
17	0.58	–0.77	–1.33	0.84	–0.5	–1.03	1.24	–0.07	–0.56	2.66	1.44	1.11	4.09	2.94	2.76	4.49	3.36	3.23	4.75	3.64	3.53
18	0.49	–0.6	–1.26	0.76	–0.31	–0.93	1.17	0.14	–0.42	2.62	1.71	1.4	4.07	3.28	3.2	4.48	3.72	3.71	4.74	4.01	4.04
19	0.49	–0.39	–1.26	0.76	–0.09	–0.9	1.18	0.39	–0.34	2.66	2.04	1.64	4.14	3.7	3.62	4.56	4.17	4.18	4.83	4.47	4.54
20	0.62	–0.21	–1.35	0.9	0.12	–0.95	1.33	0.62	–0.33	2.87	2.39	1.86	4.4	4.16	4.04	4.83	4.66	4.65	5.11	4.99	5.06
21	0.81	–0.02	–1.42	1.11	0.35	–0.97	1.56	0.89	–0.29	3.17	2.81	2.14	4.78	4.72	4.55	5.23	5.27	5.23	5.53	5.62	5.68
22	1.14	0.31	–1.44	1.45	0.69	–0.95	1.94	1.29	–0.19	3.64	3.38	2.47	5.35	5.47	5.13	5.83	6.07	5.88	6.15	6.45	6.37
23	1.6	0.74	–1.38	1.94	1.16	–0.84	2.46	1.81	–0.02	4.3	4.09	2.91	6.15	6.37	5.82	6.67	7.02	6.65	7.01	7.44	7.19
24	2.14	1.19	–1.2	2.51	1.64	–0.61	3.09	2.34	0.3	5.11	4.82	3.47	7.14	7.3	6.64	7.71	8	7.54	8.08	8.46	8.12
25	2.67	1.58	–0.89	3.09	2.07	–0.26	3.72	2.82	0.71	5.97	5.48	4.12	8.22	8.14	7.52	8.85	8.9	8.49	9.27	9.39	9.11
26	3.08	1.9	–0.48	3.54	2.42	0.19	4.24	3.22	1.2	6.73	6.04	4.78	9.22	8.86	8.37	9.92	9.66	9.38	10.38	10.18	10.04
27	3.27	2.11	–0.07	3.77	2.66	0.62	4.54	3.5	1.67	7.24	6.47	5.36	9.95	9.44	9.05	10.72	10.29	10.09	11.21	10.83	10.77
28	3.31	2.27	0.32	3.84	2.84	1.02	4.65	3.72	2.08	7.5	6.83	5.85	10.36	9.95	9.62	11.17	10.83	10.68	11.69	11.4	11.38
29	3.47	2.32	0.59	4.02	2.91	1.3	4.86	3.83	2.38	7.84	7.07	6.21	10.81	10.31	10.03	11.65	11.23	11.12	12.2	11.82	11.82
30	3.83	2.35	0.77	4.4	2.97	1.49	5.27	3.92	2.58	8.34	7.27	6.45	11.4	10.63	10.32	12.27	11.58	11.42	12.84	12.2	12.13
31	4.23	2.46	0.96	4.81	3.1	1.68	5.69	4.09	2.79	8.83	7.57	6.71	11.96	11.06	10.63	12.84	12.04	11.74	13.42	12.69	12.46
32	4.55	2.64	1.17	5.14	3.31	1.91	6.04	4.34	3.04	9.23	7.99	7.04	12.42	11.63	11.03	13.33	12.66	12.17	13.91	13.33	12.9
33	4.79	2.88	1.37	5.39	3.58	2.13	6.32	4.67	3.3	9.59	8.5	7.43	12.86	12.33	11.55	13.79	13.42	12.72	14.39	14.12	13.48
34	4.97	3.15	1.56	5.59	3.9	2.35	6.55	5.04	3.57	9.95	9.08	7.88	13.34	13.12	12.19	14.31	14.27	13.41	14.93	15.01	14.2
35	5.08	3.43	1.72	5.74	4.22	2.55	6.75	5.43	3.84	10.34	9.69	8.38	13.93	13.95	12.92	14.95	15.15	14.21	15.61	15.94	15.04
36	5.11	3.72	1.86	5.82	4.54	2.75	6.92	5.81	4.11	10.79	10.27	8.9	14.66	14.74	13.7	15.76	16.01	15.06	16.47	16.83	15.94
37	5.05	4	2.02	5.83	4.86	2.95	7.04	6.17	4.38	11.31	10.82	9.41	15.57	15.46	14.44	16.78	16.78	15.87	17.57	17.63	16.79
38	4.84	4.28	2.24	5.73	5.16	3.2	7.09	6.51	4.67	11.91	11.27	9.86	16.72	16.03	15.04	18.08	17.38	16.51	18.97	18.26	17.47
39	4.45	4.55	2.53	5.47	5.43	3.49	7.03	6.79	4.98	12.57	11.58	10.21	18.1	16.36	15.45	19.67	17.72	16.94	20.69	18.6	17.9
40	3.85	4.76	2.87	5.02	5.64	3.83	6.82	6.98	5.3	13.18	11.74	10.48	19.54	16.49	15.67	21.34	17.84	17.14	22.51	18.72	18.1
41	3.1	4.96	3.19	4.44	5.83	4.13	6.5	7.17	5.58	13.77	11.88	10.7	21.04	16.59	15.82	23.11	17.93	17.27	24.44	18.8	18.21
42	2.19	5.18	3.51	3.72	6.04	4.44	6.07	7.36	5.87	14.37	12.03	10.91	22.67	16.69	15.96	25.02	18.02	17.39	26.55	18.87	18.32

The GAMLSS model was used to calculate centiles. The sample sizes for the categories of underweight, normal and overweight or obese are 66, 375 and 199, respectively. Uw, underweight (BMI < 18.5), N, normal (BMI ≥ 18.5 and <25), Ow/Ob, overweight/obese (BMI ≥ 25).

### Comparison of gestational weight gain with the Intergrowth-21st reference

**FIGURE 4:**
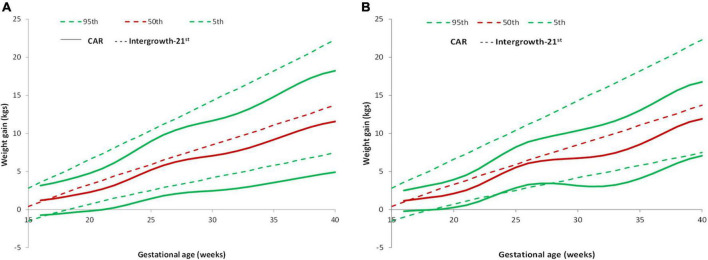
Pattern of GWG and their comparison with Intergrowth-21st standard. **(A)** Comparison of GWG curves of the REVAMP cohort (*n* = 640) with Intergrowth-21st by gestational age. **(B)** Comparison of GWG curves of the REVAMP cohort low-risk population (*n* = 102) with Intergrowth-21st reference.

The 5, 50, and 95th percentiles for GWG in the REVAMP cohort (total sample) and the low-risk group from this cohort were compared with the Intergrowth-21st reference (Figures 4A,B). The participants gained less weight throughout pregnancy compared to Intergrowth-21st and this difference was more pronounced in later pregnancy and in the higher centiles.

Intergrowth–21st prescribes an average cumulative GWG of 7.47 kg at 28 weeks, 9.52 kg at 32 weeks, 11.58 kg at 36 weeks, and 13.69 kg at 40 weeks. In the REVAMP study, we observed an average cumulative GWG of 6.6 kg at 28 weeks, 7.68 kg at 32 weeks, 9.75 kg at 36 weeks, and 11.56 kg at 40 weeks (Table 2). The percentage of women with GWG < 10th centile at delivery was similar, both in the total sample (25.3%) and the low risk group (24.5%) (Table 4).

**TABLE 4:** Comparison of GWG in the REVAMP cohort and low risk population with the Intergrowth 21st reference.

Gestational age	*n*	Less than 5th centile % (95% CI)	Less than 10th centile % (95% CI)	More than 95th centile % (95% CI)
**Total sample**				
18–22	535	10.8 (8.9, 12.9)	23.4 (20.8, 25.9)	1.1 (0.5, 1.8)
26–28	533	11.4 (9.4, 13.4)	18.0 (15.6, 20.5)	1.9 (1.1, 2.6)
At delivery	538	14.9 (12.6, 17.2)	25.3 (22.6, 27.9)	1.7 (0.9, 2.4)
**Low risk population**				
18–22	102	15.7 (9.5, 22.6)	23.5 (15.5, 31.5)	0
26–28	102	11.8 (6.0, 17.8)	15.7 (9.2, 23.3)	1.0 (0, 3.2)
At delivery	102	11.8 (5.9, 17.9)	24.5 (17.1, 32.8)	0

Total sample-pregnant women who fulfilled the selection criteria of the REVAMP study. Low risk population-pregnant women who are free from any pregnancy complications (selection criteria similar to Intergrowth–21st).

### Comparison of gestational weight gain curve with the GARBH-Ini cohort

**FIGURE 5:**
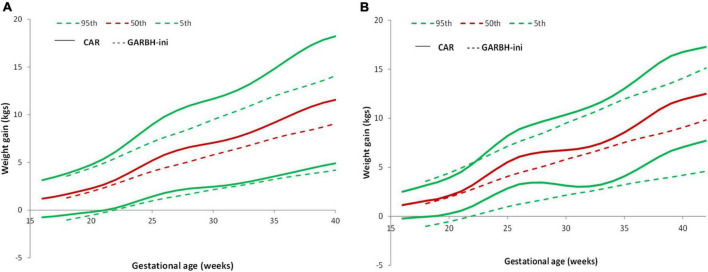
Pattern of GWG and their comparison with GARBH-Ini cohort. **(A)** Comparison of GWG curves of the REVAMP cohort (*n* = 640) with GARBH-Ini by gestational age. **(B)** Comparison of GWG curves of the REVAMP cohort low-risk population (*n* = 102) with GARBH-Ini reference.

The study participants in the total sample and the low-risk group gained more weight as compared to those in the GARBH-Ini cohort (Figures 5A,B). GARBH-Ini documented an average cumulative GWG of 5.07 kg (28 weeks), 6.45 kg (32 weeks), 7.86 kg (36 weeks), and 9.06 kg (40 weeks) (24). In the REVAMP study, we observed a higher average cumulative GWG; 6.6 kg (28 week), 7.68 kg (32 week), 9.75 kg (36 week), and 11.56 kg (40 week).

In comparison to the GARBH–Ini cohort centile curves, our study participants gained more weight in all the BMI categories (24).

### Maternal height and gestational weight gain

We also compared GWG data in relation to maternal height. Supplementary Table 4 presents 3rd, 5, 50, 90, and 97th centiles for gestational weight gain in short (<153 cm) and tall women (>153 cm).

### Association between antenatal risk factors and total gestational weight gain

A multivariable regression analysis was undertaken to examine the association of various factors such as age, BMI, height, parity, socio-economic status, family type, cooking fuel, occupation, drinking water, type of house and hemoglobin with the total GWG at delivery (Table 5). There was a 127 g reduction in GWG for every unit (kg/m^2^) increase in first trimester BMI. Reports indicate that maternal socioeconomic status is an important in determining maternal health (41). Family type, cooking fuel, occupation, and drinking water, type of house, socioeconomic status and religion were not related to GWG. In addition, we found moderate physical activity (*p* = 0.11) and dietary score (*p* = 0.52) at recruitment were not associated with total GWG.

**TABLE 5:** Association between antenatal factors and total GWG evaluated in a multivariable regression analysis (*n* = 640).

Antenatal risk factor	Regression coefficient	Standard error	*P*-value
Age (years)	−0.11296	0.049	0.022
First trimester BMI (kg/m^3^)	−0.12765	0.045	0.004
Height (cm)	0.08063	0.029	0.006
Parity: Nulliparous	Referent		
Parity: Multiparous	−0.85262	0.399	0.033
Religion: Non-Hindu	Referent		
Religion: Hindu	0.04104	0.727	0.955
Type of family: Nuclear	Referent		
Type of family: Non- nuclear	0.02657	0.362	0.942
Clean fuel (LPG/Electricity)	1.13338	1.393	0.416
Piped/bottled drinking water	0.28108	0.663	0.672
Type of the house: Pucca (Engineered)	Referent		
Type of the house—Kutcha (Non-engineered)	−2.07424	1.361	0.128
Type of the house-Semi	−0.04408	0.764	0.954
Occupation: Unemployed	Referent		
Occupation: Employed	0.35567	0.389	0.362
Socio-economic status: Lower class	Referent		
Socio-economic status: Upper class	0.26197	0.475	0.581
Hemoglobin (g%)	−0.42854	0.144	0.003
Dietary score	0.001	0.001	0.526
Moderate—Physical activity	0.003	0.001	0.107
Intercept	1.448867	4.611	0.754

## Discussion

The current study describes the GWG among pregnant women having ante-natal care at two hospitals in Pune, Western India. In this longitudinal follow-up cohort study, we derived trimester specific reference centiles of GWG and compared them with the Intergrowth–21st reference as well as with a cohort from Northern India (GARBH-Ini). Intergrowth-21st project is the first study, to report GWG from 15–40 weeks of pregnancy in normal weight women, using data from 8 countries and it also includes three Asian countries (India, China, and Oman) (9). The Intergrowth study recruited healthy well-nourished women to arrive at recommendations for optimal weight gain. The Indian participants in Intergrowth-21st were from Nagpur, central India (*N* = 455). The GWG in the Nagpur women was lower as compared to women enrolled from other countries (30). The Intergrowth-21 study concluded that despite the range of cultures, behaviors, clinical practices, and traditions, patterns of gestational weight gain are similar in populations. Subsequently, GWG in Northern India women was reported to be significantly lower than the Intergrowth-21st reference (24), which raised questions on the routine using of these references to diagnose appropriate GWG in the Indian context. The current study also confirms that the GWG is lower in comparison to the Intergrowth–21st reference.

Underweight women had greater GWG than normal and overweight women. Similar findings have been reported from Italy and India (24, 42). The observed 127 g reduction in GWG per unit increase in first trimester BMI is similar to the study from Northern India (24). During pregnancy, fat is stored to secure an energy supply for fetal growth and lactation. A prospective study by Zanardo et al., reports that in obese women, no additional storage is necessary, and hence pregnancy weight gain could be restricted (43).

Earlier Asian studies on GWG were either cross-sectional or compared GWG with the IOM, 2009 guidelines. IOM guidelines are appropriate for American women, and are based on pre-pregnancy BMI, singleton pregnancies, primigravida mothers of high social status and those with no physical activity (15). Earlier Indian studies also reported inadequate GWG in comparison to the IOM reference (17–19, 42). The observed differences from IOM and Intergrowth may be due ethnicity, lifestyle, and nutritional factors.

The higher GWG in comparison to GARBH–Ini cohort may reflect the better socioeconomic status of women from our study. The majority of GARBH–Ini participants belonged to the upper lower class while our cohort is predominantly constituted by the upper class. The geographical location may also have contributed since the diet patterns vary between western and northern regions of India. International GWG curves are not classified based on BMI (9). The data from our study provides a reference chart for GWG in different BMI categories, which will help clinicians to monitor GWG during pregnancy according to BMI category.

The greater GWG in pregnant women above 153 cm in height is in conformity with earlier findings (44). The association of total GWG with maternal age, BMI, parity, and height has also been reported earlier (24, 45, 46). In addition, we observed, higher hemoglobin concentration were associated with lower GWG. Higher hemoglobin may be linked with low plasma volume expansion which may in turn lead to lower gestational weight gain.

The strength of our study includes the prospective design, accurate gestational age measurements, and multiple antenatal weight measurements which allow us to assess trimester-specific GWG. Our study has some limitations. Firstly, the initial recruitment was done in the first trimester (11–14 weeks) and the weight at this visit was approximated as the pre-conception metric. Measured weight in early pregnancy provides a reasonable quantification of pre-pregnancy weight and is used for calculating the pre-pregnancy BMI and GWG (47, 48). It has been reported that mean differences between self-reported preconception weight and measured first-trimester weights was below 2 kg, and had little impact on BMI classification and GWG calculation (48). All women included in this study are from an urban area who have better access to healthcare, and a higher educational level than rural Indian women, and did not include women from rural area. Also, for some gestational weeks, the sample size is very low.

## Conclusion

In conclusion, this study provides GWG charts from “healthy” pregnant women of upper socio-economic status from an urban setting in Pune, Western, India, which confirms that the GWG of Indian women is lower than the prescriptive standards of the Intergrowth charts. These charts would be appropriate for routine obstetric use in India, particularly the Western region, to prevent misclassification errors with the available Intergrowth-21st and IOM references.

## Data availability statement

The study data will be available on request subject to approval from the Institutional Ethics Committee and Health Ministry Screening Committee, Government of India.

## Ethics statement

The studies involving human participants were reviewed and approved by Institutional Ethics Committee, Bharati Vidyapeeth Deemed University, Pune. The patients/participants provided their written informed consent to participate in this study.

## Author contributions

HS and SJ contributed to study conception and design. KD, HP, VK, and SGun contributed to data collection. HS, SM, and KR contributed to statistical analysis and interpretation of results. KD, SJ, SGup, GW, SL, NC, BK, HS, and CF contributed to draft manuscript preparation. All authors reviewed the manuscript and approved the submitted version.
