# Supplementary material for: Gestational weight gain in the REVAMP pregnancy cohort in Western India: Comparison with international and national references
Source: Front Med (Lausanne). 2022 Oct 5;9:1022990. doi: 10.3389/fmed.2022.1022990 (PMC9579320; doi:10.3389/fmed.2022.1022990)
Supplement: Supplementary file 1 [file Table_1.docx]

**Supplementary Table 1: Comparison of selection criteria used to identify low risk study participants in Intergrowth-21st and REVAMP cohort**

| **Intergrowth-21st** | **REVAMP cohort** |
| --- | --- |
| Aged ≥18 and <35 years | Aged ≥18 and <35 years |
| Body mass index ≥18.5 and <30 kg/m^2^ | Body mass index ≥18.5 and <30 kg/m^2^ |
| Height ≥153 cm | Height ≥153 cm |
| Singleton pregnancy | Singleton pregnancy |
| A known last menstrual period with regular cycles (defined as 28 ± 4 days) without hormonal contraceptive use, or breastfeeding in the months before pregnancy | Regular menstrual cycle |
| Natural conception | Natural conception |
| No relevant past medical history, with no need for long-term medication (including fertility treatment and over-the-counter medicines, but excluding routine iron, folate, calcium, iodine or multivitamin supplements). | No history of chronic illnesses: hypertension, diabetes |
| No evidence of socioeconomic constraints likely to impede fetal growth identified using local definitions of social risk | Middle and upper socioeconomic status |
| No use of tobacco or recreational drugs such as cannabis in the 3 months before or after becoming pregnant. | No reported history of use of tobacco or substance abuse |
| No heavy alcohol use (defined as >5 units  (50 ml pure alcohol) per week) since becoming pregnant. | No reported history of use of alcohol |
| No more than one miscarriage in the two previous consecutive pregnancies. | No more than one miscarriages in the prior pregnancies |
| No previous baby delivered preterm (<37+0 weeks of gestation) or with a birth weight <2500 g or >4500 g. | No history of preterm birth or low birth weight in past pregnancies |
| No previous neonatal or fetal death, previous baby with any congenital malformations, and no evidence in present pregnancy of congenital disease or fetal anomaly. | No history of stillbirth in past pregnancies; congenital anomalies in the present pregnancy |
| No previous pregnancy affected by pre-eclampsia/eclampsia, HELLP syndrome or a related pregnancy-associated condition. | No previous pregnancy affected by pre- eclampsia/eclampsia |
| No clinically significant atypical red cell alloantibodies. | No history of Rh incompatibility in the past pregnancy |
| Negative urinalysis | Negative urinalysis |
| Systolic blood pressure <140 mmHg and diastolic blood pressure <90 mmHg. | Systolic blood pressure <140 mmHg and diastolic blood pressure <90 mmHg |
| No diagnosis or treatment for anaemia during this pregnancy (hemoglobin levels will be monitored throughout pregnancy). | Hemoglobin ≥11 g/dl |
| No clinical evidence of any other sexually transmitted diseases, including syphilis and clinical trichomoniasis. | No clinical evidence of syphilis or HIV or HBsAg |
| Not in an occupation with risk of exposure to chemicals or toxic substances, or very physically demanding activity to be evaluated by local standards. Also women should not be conducting vigorous or contact sports, such as scuba diving or similar activities. | Use of clean fuel, no exposure to passive smoking, routine household or sedentary job |
