# Supplementary material for: Gestational weight gain in the REVAMP pregnancy cohort in Western India: Comparison with international and national references
Source: Front Med (Lausanne). 2022 Oct 5;9:1022990. doi: 10.3389/fmed.2022.1022990 (PMC9579320; doi:10.3389/fmed.2022.1022990)
Supplement: Supplementary file 2 [file Table_2.DOCX]

**Supplementary Table 2: GWG (in kg) percentiles at various gestational ages (in weeks) in the low-risk population (n = 102)**

| **Gestational age**  **(weeks)** | **Centiles for GWG (kg)** | | | | | | |
| --- | --- | --- | --- | --- | --- | --- | --- |
|  | **3^rd^** | **5^th^** | **25^th^** | **50^th^** | **75^th^** | **95^th^** | **97^th^** |
| 16 | -0.85 | -0.6 | 0.43 | 1.15 | 1.86 | 1.86 | 1.86 |
| 17 | -0.81 | -0.54 | 0.58 | 1.36 | 2.13 | 2.13 | 2.13 |
| 18 | -0.79 | -0.49 | 0.73 | 1.57 | 2.41 | 2.41 | 2.41 |
| 19 | -0.76 | -0.45 | 0.86 | 1.76 | 2.67 | 2.67 | 2.67 |
| 20 | -0.6 | -0.26 | 1.14 | 2.1 | 3.06 | 3.06 | 3.06 |
| 21 | -0.37 | -0.01 | 1.5 | 2.53 | 3.57 | 3.57 | 3.57 |
| 22 | 0.04 | 0.43 | 2.06 | 3.19 | 4.31 | 4.31 | 4.31 |
| 23 | 0.58 | 1.01 | 2.77 | 4.00 | 5.22 | 5.22 | 5.22 |
| 24 | 1.12 | 1.58 | 3.48 | 4.8 | 6.12 | 6.12 | 6.12 |
| 25 | 1.6 | 2.09 | 4.12 | 5.53 | 6.94 | 6.94 | 6.94 |
| 26 | 1.95 | 2.47 | 4.6 | 6.08 | 7.56 | 7.56 | 7.56 |
| 27 | 2.05 | 2.59 | 4.82 | 6.37 | 7.92 | 7.92 | 7.92 |
| 28 | 1.98 | 2.55 | 4.92 | 6.56 | 8.2 | 8.2 | 8.2 |
| 29 | 1.74 | 2.36 | 4.9 | 6.66 | 8.43 | 8.43 | 8.43 |
| 30 | 1.44 | 2.11 | 4.85 | 6.75 | 8.65 | 8.65 | 8.65 |
| 31 | 1.22 | 1.93 | 4.85 | 6.87 | 8.9 | 8.9 | 8.9 |
| 32 | 1.16 | 1.9 | 4.96 | 7.09 | 9.22 | 9.22 | 9.22 |
| 33 | 1.26 | 2.04 | 5.22 | 7.44 | 9.65 | 9.65 | 9.65 |
| 34 | 1.55 | 2.35 | 5.64 | 7.93 | 10.21 | 10.21 | 10.21 |
| 35 | 1.99 | 2.81 | 6.2 | 8.55 | 10.9 | 10.9 | 10.9 |
| 36 | 2.56 | 3.4 | 6.87 | 9.29 | 11.7 | 11.7 | 11.7 |
| 37 | 3.2 | 4.07 | 7.62 | 10.09 | 12.56 | 12.56 | 12.56 |
| 38 | 3.85 | 4.73 | 8.36 | 10.88 | 13.4 | 13.4 | 13.4 |
| 39 | 4.41 | 5.3 | 8.96 | 11.51 | 14.06 | 14.06 | 14.06 |
| 40 | 4.79 | 5.69 | 9.36 | 11.91 | 14.46 | 14.46 | 14.46 |
| 41 | 5.13 | 6.02 | 9.67 | 12.21 | 14.75 | 14.75 | 14.75 |
| 42 | 5.47 | 6.35 | 9.97 | 12.49 | 15.01 | 15.01 | 15.01 |

The GAMLSS was used to calculate centiles.
