# Supplementary material for: Gestational weight gain in the REVAMP pregnancy cohort in Western India: Comparison with international and national references
Source: Front Med (Lausanne). 2022 Oct 5;9:1022990. doi: 10.3389/fmed.2022.1022990 (PMC9579320; doi:10.3389/fmed.2022.1022990)
Supplement: Supplementary file 3 [file Table_3.docx]

**Supplementary Table 3: Total GWG at different first trimester BMI categories**

| **Categories compared** | **Difference in GWG (kg)** | **Range of difference in GWG (kg)** | **p value** |
| --- | --- | --- | --- |
| Normal weight –  Underweight | -0.98 | -0.26; 2.22 | 0.152 |
| Overweight/ Obese –  Underweight | -2.40 | -1.09; -3.75 | <0.001 |
| Overweight/ Obese –  Normal weight | -1.43 | -2.24; -0.61 | <0.001 |

P value: <0.001; ANOVA was used for statistical analysis
