# Supplementary material for: Gestational weight gain in the REVAMP pregnancy cohort in Western India: Comparison with international and national references
Source: Front Med (Lausanne). 2022 Oct 5;9:1022990. doi: 10.3389/fmed.2022.1022990 (PMC9579320; doi:10.3389/fmed.2022.1022990)
Supplement: Supplementary file 4 [file Table_4.docx]

**Supplementary Table 4: 3^rd^, 5^th^, 50^th^, 95^th^ and 97^th^ centiles for gestational weight gain in**

**short and tall women**

| **Gestational age (wks)/ Height (cm)** | **3^rd^**  **Centile** | | **5^th^**  **Centile** | | **50^th^**  **Centile** | | **95^th^**  **Centile** | | **97^th^**  **Centile** | |
| --- | --- | --- | --- | --- | --- | --- | --- | --- | --- | --- |
|  | <153 | ≥153 | <153 | ≥153 | <153 | ≥153 | <153 | ≥153 | <153 | ≥153 |
| 17 | -1.43 | -0.74 | -0.62 | -0.02 | 1.12 | 1.52 | 2.85 | 3.05 | 3.66 | 3.76 |
| 18 | -1.11 | -0.65 | -0.26 | 0.12 | 1.55 | 1.74 | 3.36 | 3.36 | 4.20 | 4.11 |
| 19 | -0.90 | -0.52 | -0.01 | 0.30 | 1.89 | 2.03 | 3.79 | 3.75 | 4.68 | 4.56 |
| 20 | -0.94 | -0.33 | 0.02 | 0.55 | 2.04 | 2.4 | 4.07 | 4.26 | 5.01 | 5.13 |
| 21 | -0.88 | -0.16 | 0.15 | 0.80 | 2.32 | 2.83 | 4.50 | 4.86 | 5.52 | 5.80 |
| 22 | -0.67 | 0.10 | 0.44 | 1.14 | 2.78 | 3.37 | 5.13 | 5.60 | 6.23 | 6.64 |
| 23 | -0.32 | 0.43 | 0.87 | 1.58 | 3.4 | 4.02 | 5.93 | 6.47 | 7.12 | 7.62 |
| 24 | 0.11 | 0.80 | 1.39 | 2.05 | 4.12 | 4.72 | 6.85 | 7.39 | 8.12 | 8.64 |
| 25 | 0.51 | 1.19 | 1.88 | 2.53 | 4.81 | 5.41 | 7.74 | 8.28 | 9.12 | 9.63 |
| 26 | 0.77 | 1.58 | 2.24 | 3.01 | 5.36 | 6.05 | 8.49 | 9.08 | 9.96 | 10.51 |
| 27 | 0.87 | 1.91 | 2.41 | 3.39 | 5.70 | 6.56 | 8.99 | 9.73 | 10.53 | 11.21 |
| 28 | 0.9 | 2.12 | 2.53 | 3.65 | 5.99 | 6.92 | 9.46 | 10.19 | 11.08 | 11.72 |
| 29 | 0.96 | 2.18 | 2.67 | 3.75 | 6.32 | 7.11 | 9.98 | 10.46 | 11.69 | 12.03 |
| 30 | 1.11 | 2.15 | 2.90 | 3.76 | 6.73 | 7.21 | 10.56 | 10.66 | 12.34 | 12.27 |
| 31 | 1.34 | 2.16 | 3.21 | 3.82 | 7.20 | 7.37 | 11.19 | 10.92 | 13.06 | 12.58 |
| 32 | 1.61 | 2.24 | 3.56 | 3.96 | 7.72 | 7.64 | 11.88 | 11.32 | 13.83 | 13.04 |
| 33 | 1.91 | 2.38 | 3.94 | 4.18 | 8.29 | 8.02 | 12.63 | 11.87 | 14.66 | 13.66 |
| 34 | 2.22 | 2.57 | 4.33 | 4.46 | 8.86 | 8.51 | 13.39 | 12.55 | 15.51 | 14.44 |
| 35 | 2.49 | 2.8 | 4.69 | 4.80 | 9.40 | 9.08 | 14.1 | 13.36 | 16.30 | 15.36 |
| 36 | 2.73 | 3.07 | 5.00 | 5.19 | 9.85 | 9.72 | 14.71 | 14.25 | 16.98 | 16.36 |
| 37 | 2.92 | 3.38 | 5.23 | 5.61 | 10.19 | 10.39 | 15.15 | 15.16 | 17.47 | 17.39 |
| 38 | 3.02 | 3.76 | 5.36 | 6.09 | 10.36 | 11.06 | 15.36 | 16.03 | 17.7 | 18.35 |
| 39 | 3.02 | 4.22 | 5.36 | 6.60 | 10.36 | 11.68 | 15.35 | 16.76 | 17.69 | 19.13 |
| 40 | 2.94 | 4.71 | 5.26 | 7.09 | 10.22 | 12.2 | 15.18 | 17.31 | 17.50 | 19.69 |
| 41 | 2.80 | 5.21 | 5.11 | 7.60 | 10.04 | 12.7 | 14.98 | 17.80 | 17.29 | 20.18 |

Centiles were estimated from the GAMLSS
